# Factors influencing HIV testing among women in Mozambique: insights from the 2022/23 demographic and health survey

**DOI:** 10.1186/s12889-026-27411-3

**Published:** 2026-04-28

**Authors:** Amr Ahmed Aly Ibrahim, Abdalrahman Salah Shehata, Sara Hosny El-Farargy, Moaz Yasser Darwish, Ahmed Reda Bahr, Karim Samir Attia, Omar Abbas, Mahmoud Shaaban Abdelgalil

**Affiliations:** 1 Faculty of Medicine, Menoufia University, Menoufia, Egypt; 2 Faculty of Medicine, Qena University, Qena, Egypt; 3 Faculty of Medicine, Benha University, Benha, Egypt; 4 Faculty of Medicine, Fayoum University, Fayoum, Egypt; 5 Faculty of Medicine, Alexandria University, Alexandria, Egypt; 6 Faculty of Medicine, Ain-shams University, Cairo, Egypt; 7 Research Insights Arab Network, Cairo, Egypt

**Keywords:** HIV Testing, Mozambique, Socio-Demographic Factors, DHS

## Abstract

**Background:**

Mozambique bears a high HIV burden, with low testing uptake among young women. This study analyzes socio-demographic and behavioral factors influencing HIV testing among women aged 15–49, using data from the 2022/23 Demographic and Health Survey (DHS).

**Methods:**

A secondary analysis of the Mozambique DHS 2023 dataset was performed using R (Version 4.4.2, R Core Team) to explore factors associated with HIV testing uptake. Variables including age, education, marital status, geographic location, and socioeconomic indicators were analyzed. Descriptive statistics were calculated, and associations were evaluated using a modified Poisson regression model with robust standard errors to estimate risk ratios. Both univariate and multivariable models were used to identify significant predictors, accounting for the survey’s complex design.

**Results:**

The analysis included 13,183 women aged 15–49 years, of whom 67.56% reported having ever been tested for HIV. Multivariate analysis revealed that older age, higher educational attainment, greater household wealth, living with a partner, recent health facility visits, and internet use were significantly associated with higher likelihood of HIV testing. Conversely, rural residence was significantly associated with lower likelihood of testing. Regional variation in testing uptake was also observed, with most provinces showing significantly higher testing rates compared to Niassa. Variables including religion, health insurance, newspaper reading, and television viewing were not independently associated with HIV testing after adjustment for other covariates (all *p* > 0.05).

**Conclusions:**

Mozambique must prioritize expanding HIV testing in rural areas by enhancing healthcare access and community outreach. Promoting awareness through digital platforms and integrating HIV education into public programs can further improve testing uptake. Strengthening education and healthcare services is vital to achieving better prevention and early treatment outcomes.

**Supplementary Information:**

The online version contains supplementary material available at 10.1186/s12889-026-27411-3.

## Introduction

Human Immunodeficiency Virus (HIV) continues to be a significant global health challenge, characterized by its profound impact on the immune system, leaving individuals vulnerable to opportunistic infections and chronic health complications [1]. In 2024, an estimated 40.8 million individuals globally were living with HIV, with annual new infections estimated at 1.3 million and a death toll nearing 630,000 [2]. Despite extensive advancements in treatment and prevention, sub-Saharan Africa remains the epicenter of the HIV epidemic, bearing the brunt of global disease burden [3].

Mozambique, as one of the hardest-hit countries, exhibits a strikingly high HIV prevalence rate, with approximately 12.4% of adults aged 15–49 years living with the virus [4]. The country is among the 10 with the highest number of new infections worldwide, making HIV a persistent public health crisis [5]. In addition to widespread community transmission, vulnerable populations such as women, adolescents, sex workers, and men who have sex with men (MSM) face disproportionate risks of infection due to intersecting social, economic, and structural inequalities [6]. Alarmingly, young women bear the greatest burden, accounting for a significant proportion of new infections due to factors such as gender inequality, early marriage, limited access to education, and sexual violence [7].

HIV testing is a critical entry point to care, enabling early diagnosis and timely initiation of antiretroviral therapy (ART), which improves individual health outcomes and reduces viral transmission at the population level [8]. Despite its importance, Mozambique faces significant barriers to testing, particularly in rural areas where access to facilities is limited. Cultural stigma, fears of discrimination, and confidentiality concerns further deter testing, while gender norms discourage men and stigmatize women for seeking care [9].

Traditional facility-based testing models have struggled to reach key populations, particularly those in remote areas or with limited mobility due to work. Community-based testing and HIV self-testing (HIVST) offer promising solutions by providing privacy and accessibility, but resource constraints, policy gaps, and limited public awareness hinder their broader implementation [10].

Mozambique lags the UNAIDS 95-95-95 targets for 2030, which aim for 95% HIV awareness, 95% ART coverage, and 95% viral suppression [8]. Reports from 2021 showed that 71.6% of adults living with HIV in the country were aware of the diagnosis, 96.4%of them were on ART and 89.4% had suppressed viral loads [11]. Addressing this gap requires a nuanced understanding of factors influencing HIV testing, particularly among young women, a high-risk demographic. This study examines the socio-demographic and behavioral determinants of HIV testing among women aged 15–49 years old in Mozambique using DHS data 2022/23, providing evidence to inform targeted interventions and support national efforts to curb the epidemic.

## Methods

### Study design and data source

This secondary analysis utilized data from the Mozambique Demographic and Health Survey (MDHS) conducted in 2022/23. The MDHS is a nationally representative cross-sectional survey that employs a two-stage stratified cluster sampling design to ensure representativeness across urban and rural areas. Ethical approval for the survey was granted by the appropriate institutional review boards, and permission to use the data was obtained from the DHS Program. The study was reported in accordance with the Strengthening the Reporting of Observational Studies in Epidemiology (STROBE) Statement, and the completed STROBE checklist is provided as supplementary material [12].

### Study population

The study population included women aged 15–49 years. For this analysis, participants were categorized into two groups based on their self-reported HIV testing status: those who had been tested for HIV and those who had never been tested. Detailed information regarding the sample size determination and sampling procedures is provided in the methodology of the MDHS [13]. As this study represents a secondary analysis of DHS data, the sample size was predetermined by the original survey design.

### Variables

The dependent variable was HIV testing status, categorized as “ever tested” or “never tested.” Independent variables included: Sociodemographic factors: age, educational attainment, wealth index and geographical region including province and type of place of residence (rural vs. urban). Behavioral factors: frequency of media exposure (radio, TV, internet), and healthcare utilization. Healthcare access: health insurance coverage and visits to healthcare facilities in the last 12 months.

### Statistical analysis

To account for the complex sampling design of the survey, we incorporated stratification, clustering, and sampling weights in the analysis following the DHS recommendations [14]. The eight-digit sample weight variable, which included six implied decimal places, was scaled by dividing the weighting factor by 1,000,000. All variables were summarized as frequencies and percentages and were compared using the Chi-square test with the Rao-Scott adjustment. To model variables associated with HIV testing, we deployed a modified Poisson regression with robust standard errors. Separate univariate models were fitted to estimate the unadjusted risk ratio (RR) for each variable. We then fitted a multivariate model including all variables (age, region, residence, education, wealth index, religion, marital status, health insurance, healthcare utilization, and media exposure) regardless of their significance in univariate analysis, to control for confounding and estimate adjusted risk ratios (aRRs).

Furthermore, we fitted another multivariate model incorporating only the core socio-demographic determinants (age, residence, education, wealth index, having ever been married or in a union, having health insurance, visiting a health facility in the last 12 months, and internet use) as a sensitivity analysis. In all multiple regression models, the likelihood ratio test (LRT) was used to assess the omnibus significance. All estimates are presented with the corresponding 95% confidence intervals (CIs). A two-sided p-value of < 0.05 was considered statistically significant. Reference groups in the regression models were selected based on variable type. For ordered categorical variables (age, education, and wealth index), the lowest category was used as the reference to assess dose–response relationships. For nominal variables (such as region and marital status), reference categories were chosen based on theoretical relevance and sample size, consistent with standard epidemiological practice and previous DHS-based studies.The analysis for this paper was conducted in R (Version 4.4.2, R Core Team), utilizing the “tidyverse”, “survey”, and “gtsummary” packages.

## Results

### Participant characteristics according to HIV testing

The study included 13,183 women in Mozambique, of whom 8,906 (62.8%) had ever been tested for HIV and 4,277 (37.2%) had not (Table 1). HIV testing was significantly associated with several sociodemographic characteristics (all *p* < 0.001).

**Table 1:** Characteristics of women included in the study according to HIV testing in Mozambique, 2022–2023

Characteristic	Ever been tested for HIV, *N* = 13 183		*p*-value^b^
	No, *n* = 4 277^*a*^	Yes, *n* = 8 906^*a*^	
Age (years)			**< 0.0001**
15–19	1 829 (42.8%)	1 221 (13.7%)	
20–24	729 (17.0%)	1 964 (22.1%)	
25–29	472 (11.0%)	1 724 (19.4%)	
30–34	319 (7.5%)	1 258 (14.1%)	
35–39	301 (7.0%)	1 185 (13.3%)	
40–44	303 (7.1%)	867 (9.7%)	
45–49	324 (7.6%)	687 (7.7%)	
Region			**< 0.0001**
Niassa	439 (10.3%)	421 (4.7%)	
Cabo Delgado	246 (5.7%)	459 (5.2%)	
Nampula	1 456 (34.0%)	1 608 (18.1%)	
Zambézia	930 (21.7%)	1 263 (14.2%)	
Tete	409 (9.6%)	905 (10.2%)	
Manica	185 (4.3%)	724 (8.1%)	
Sofala	229 (5.4%)	680 (7.6%)	
Inhambane	83 (1.9%)	472 (5.3%)	
Gaza	80 (1.9%)	590 (6.6%)	
Maputo	149 (3.5%)	1 198 (13.4%)	
Cidade de Maputo	70 (1.6%)	585 (6.6%)	
Place of residence			**< 0.0001**
Urban	1 033 (24.2%)	4 087 (45.9%)	
Rural	3 244 (75.8%)	4 819 (54.1%)	
Highest educational level			**< 0.0001**
No education	1 636 (38.2%)	1 886 (21.2%)	
Primary	1 828 (42.7%)	3 773 (42.4%)	
Secondary	799 (18.7%)	2 909 (32.7%)	
Higher	14 (0.3%)	338 (3.8%)	
Religion			**< 0.0001**
No religion	330 (7.7%)	597 (6.7%)	
Anglican	68 (1.6%)	178 (2.0%)	
Evangelical/Pentecostal	779 (18.2%)	2 942 (33.0%)	
Zion	384 (9.0%)	1 210 (13.6%)	
Islamic	1 186 (27.7%)	1 529 (17.2%)	
Catholic	1 517 (35.5%)	2 402 (27.0%)	
Other	13 (0.3%)	48 (0.5%)	
Wealth index			**< 0.0001**
Poorest	1 226 (28.7%)	1 194 (13.4%)	
Poorer	1 031 (24.1%)	1 332 (15.0%)	
Middle	856 (20.0%)	1 516 (17.0%)	
Richer	658 (15.4%)	2 152 (24.2%)	
Richest	506 (11.8%)	2 713 (30.5%)	
Ever been married or in a union			**< 0.0001**
Currently married/Lives with a man	2 296 (53.7%)	6 191 (69.5%)	
Formerly married	220 (5.2%)	434 (4.9%)	
Lived with a man	183 (4.3%)	963 (10.8%)	
No	1 578 (36.9%)	1 319 (14.8%)	
Has Health Insurance (yes)	10 (0.2%)	178 (2.0%)	**< 0.0001**
Visited Health Facility in the Last 12 Months (yes)	1 555 (36.4%)	6 040 (67.8%)	**< 0.0001**
Reads a newspaper or magazine			**< 0.0001**
Not at all	4 113 (96.2%)	7 947 (89.2%)	
Less than once a week	94 (2.2%)	570 (6.4%)	
At least once a week	70 (1.6%)	389 (4.4%)	
Listens to the radio			**< 0.0001**
Not at all	3 135 (73.3%)	5 718 (64.2%)	
Less than once a week	588 (13.8%)	1 394 (15.7%)	
At least once a week	554 (12.9%)	1 794 (20.1%)	
Watches television			**< 0.0001**
Not at all	3 306 (77.3%)	4 990 (56.0%)	
Less than once a week	270 (6.3%)	802 (9.0%)	
At least once a week	700 (16.4%)	3 115 (35.0%)	
Uses internet			**< 0.0001**
Not at all	3 989 (93.3%)	6 731 (75.6%)	
Less than once a week	21 (0.5%)	154 (1.7%)	
At least once a week	73 (1.7%)	424 (4.8%)	
Almost every day	194 (4.5%)	1 596 (17.9%)	

^*a*^ n (%)

^*b*^ Pearson’s Chi-square test: Rao & Scott adjustment

Testing patterns varied by age. Women aged 20–29 years represented the largest proportion of those who had been tested, whereas adolescents aged 15–19 years accounted for the highest proportion of women who had never been tested (42.8%). Geographic differences were also observed. Nampula contributed the largest share of women who had not been tested (34.0%), while Maputo had the highest proportion of women who had undergone HIV testing (13.4%).

Differences were also evident by place of residence and socioeconomic status. Women living in urban areas were more likely to have been tested than those residing in rural areas (45.9% vs. 24.2%). Similarly, HIV testing was more common among women with higher levels of education and among those in the richest wealth quintile.

Healthcare access and marital status were also associated with HIV testing. A larger proportion of women who had been tested were currently married or living with a partner (69.5%), and most had visited a health facility in the past 12 months (67.8%). In addition, women who had undergone HIV testing reported greater exposure to media and more frequent internet use compared with those who had never been tested.

Further details on the distribution of participant characteristics according to HIV testing status are presented in Table 1.

### Predictors of HIV testing

In the multivariate regression analysis, several sociodemographic and healthcare-related factors were independently associated with HIV testing uptake among women aged 15–49 in Mozambique (Table 2).

**Table 2:** Univariate and multivariate modified Poisson regression analysis of factors associated with HIV testing uptake among women aged 15–49 years in Mozambique

Variable	Univariate regression			Multivariate regression			
	RR^a^	95% CI^a^	*p*-value	aRR^a^	95% CI^a^	*p*-value	LRT *p*-value
Age (years)							**< 0.0001**
15–19	—	—		—	—		
20–24	1.82	1.70 to 1.95	**< 0.0001**	1.56	1.46 to 1.67	**< 0.0001**	
25–29	1.96	1.84 to 2.09	**< 0.0001**	1.61	1.50 to 1.73	**< 0.0001**	
30–34	1.99	1.87 to 2.13	**< 0.0001**	1.59	1.47 to 1.71	**< 0.0001**	
35–39	1.99	1.88 to 2.12	**< 0.0001**	1.61	1.50 to 1.72	**< 0.0001**	
40–44	1.85	1.73 to 1.98	**< 0.0001**	1.54	1.43 to 1.66	**< 0.0001**	
45–49	1.70	1.57 to 1.83	**< 0.0001**	1.48	1.37 to 1.60	**< 0.0001**	
Region							**< 0.0001**
Niassa	—	—		—	—		
Cabo Delgado	1.33	1.20 to 1.47	**< 0.0001**	1.23	1.12 to 1.35	**< 0.0001**	
Nampula	1.07	0.96 to 1.20	0.22	1.08	0.98 to 1.19	0.10	
Zambézia	1.18	1.03 to 1.34	**0.015**	1.23	1.08 to 1.39	**0.0015**	
Tete	1.41	1.28 to 1.55	**< 0.0001**	1.36	1.23 to 1.50	**< 0.0001**	
Manica	1.63	1.49 to 1.78	**< 0.0001**	1.43	1.30 to 1.58	**< 0.0001**	
Sofala	1.53	1.39 to 1.68	**< 0.0001**	1.35	1.23 to 1.49	**< 0.0001**	
Inhambane	1.74	1.60 to 1.89	**< 0.0001**	1.50	1.36 to 1.65	**< 0.0001**	
Gaza	1.80	1.66 to 1.95	**< 0.0001**	1.54	1.40 to 1.70	**< 0.0001**	
Maputo	1.82	1.67 to 1.98	**< 0.0001**	1.45	1.32 to 1.60	**< 0.0001**	
Cidade de Maputo	1.82	1.68 to 1.98	**< 0.0001**	1.43	1.30 to 1.57	**< 0.0001**	
Place of residence							**< 0.0001**
Urban	—	—		—	—		
Rural	0.75	0.72 to 0.78	**< 0.0001**	0.88	0.84 to 0.92	**< 0.0001**	
Highest educational level							**< 0.0001**
No education	—	—		—	—		
Primary	1.26	1.19 to 1.33	**< 0.0001**	1.18	1.12 to 1.23	**< 0.0001**	
Secondary	1.46	1.37 to 1.56	**< 0.0001**	1.29	1.22 to 1.36	**< 0.0001**	
Higher	1.79	1.68 to 1.91	**< 0.0001**	1.33	1.24 to 1.42	**< 0.0001**	
Wealth index							**< 0.0001**
Poorest	—	—		—	—		
Poorer	1.14	1.05 to 1.25	**0.0033**	1.10	1.01 to 1.19	**0.027**	
Middle	1.30	1.17 to 1.43	**< 0.0001**	1.15	1.05 to 1.25	**0.0027**	
Richer	1.55	1.42 to 1.70	**< 0.0001**	1.24	1.13 to 1.35	**< 0.0001**	
Richest	1.71	1.57 to 1.86	**< 0.0001**	1.16	1.05 to 1.27	**0.0023**	
Religion							0.71
No religion	—	—		—	—		
Anglican	1.12	1.01 to 1.25	**0.031**	1.02	0.92 to 1.13	0.72	
Evangelical/Pentecostal	1.23	1.15 to 1.32	**< 0.0001**	1.03	0.96 to 1.10	0.40	
Zion	1.18	1.10 to 1.27	**< 0.0001**	1.00	0.93 to 1.07	0.93	
Islamic	0.87	0.80 to 0.95	**0.0019**	1.02	0.92 to 1.12	0.75	
Catholic	0.95	0.88 to 1.03	0.20	1.03	0.95 to 1.11	0.46	
Other	1.22	1.05 to 1.43	**0.011**	1.14	0.97 to 1.35	0.11	
Ever been married or in a union							**< 0.0001**
No	—	—		—	—		
Formerly married	1.46	1.31 to 1.62	**< 0.0001**	1.39	1.26 to 1.55	**< 0.0001**	
Lived with a man	1.85	1.74 to 1.96	**< 0.0001**	1.45	1.36 to 1.54	**< 0.0001**	
Currently married/Lives with a man	1.60	1.51 to 1.70	**< 0.0001**	1.44	1.35 to 1.53	**< 0.0001**	
Has health insurance							0.38
No	—	—		—	—		
Yes	1.41	1.35 to 1.47	**< 0.0001**	0.98	0.94 to 1.03	0.38	
Visited a health facility in the last 12 months							**< 0.0001**
No	—	—		—	—		
Yes	1.55	1.48 to 1.63	**< 0.0001**	1.21	1.16 to 1.26	**< 0.0001**	
Reads a newspaper or magazine							
Not at all	—	—		—	—		0.99
Less than once a week	1.30	1.25 to 1.35	**< 0.0001**	1.00	0.96 to 1.04	0.96	
At least once a week	1.29	1.23 to 1.35	**< 0.0001**	1.00	0.95 to 1.04	0.88	
Listens to the radio							**0.043**
Not at all	—	—		—	—		
Less than once a week	1.09	1.01 to 1.17	**0.023**	0.94	0.89 to 0.99	**0.031**	
At least once a week	1.18	1.13 to 1.23	**< 0.0001**	0.98	0.95 to 1.02	0.33	
Watches television							0.63
Not at all	—	—		—	—		
Less than once a week	1.24	1.18 to 1.31	**< 0.0001**	1.02	0.97 to 1.07	0.38	
At least once a week	1.36	1.30 to 1.41	**< 0.0001**	1.01	0.97 to 1.05	0.54	
Uses internet							**0.0004**
Not at all	—	—		—	—		
Less than once a week	1.40	1.31 to 1.50	**< 0.0001**	1.10	1.03 to 1.16	**0.0031**	
At least once a week	1.36	1.30 to 1.42	**< 0.0001**	1.01	0.98 to 1.05	0.47	
Almost every day	1.42	1.37 to 1.47	**< 0.0001**	1.05	1.02 to 1.07	**0.0009**	

^a^  *RR*   Risk Ratio, *aRR*  adjusted Risk Ratio, *CI*  Confidence Interval, *LRT*  Likelihood Ratio Test

#### Age

Age was significantly associated with HIV testing (LRT *p* < 0.0001). Compared with women aged 15–19 years, all older age groups had significantly higher likelihood of testing: aged 20–24 (aRR 1.56, 95% CI: 1.46–1.67), 25–29 (aRR 1.61, 95% CI: 1.50–1.73), 30–34 (aRR 1.59, 95% CI: 1.47–1.71), 35–39 (aRR 1.61, 95% CI: 1.50–1.72), 40–44 (aRR 1.54, 95% CI: 1.43–1.66), and 45–49 (aRR 1.48, 95% CI: 1.37–1.60), all *p* < 0.0001.

#### Region

Region was significantly associated with testing (LRT *p* < 0.0001). Compared with Niassa province, significantly higher testing rates were observed in Cabo Delgado (aRR 1.23, 95% CI: 1.12–1.35), Zambézia (aRR 1.23, 95% CI: 1.08–1.39), Tete (aRR 1.36, 95% CI: 1.23–1.50), Manica (aRR 1.43, 95% CI: 1.30–1.58), Sofala (aRR 1.35, 95% CI: 1.23–1.49), Inhambane (aRR 1.50, 95% CI: 1.36–1.65), Gaza (aRR 1.54, 95% CI: 1.40–1.70), Maputo Province (aRR 1.45, 95% CI: 1.32–1.60), and Cidade de Maputo (aRR 1.43, 95% CI: 1.30–1.57), all *p* < 0.0001 or *p* = 0.0015. Nampula did not differ significantly from Niassa (*p* = 0.10).

#### Place of residence

Rural residence was associated with significantly lower HIV testing compared to urban residence (aRR 0.88, 95% CI: 0.84–0.92, *p* < 0.0001).

#### Education

Higher educational attainment was positively and significantly associated with HIV testing (LRT *p* < 0.0001). Compared with women with no education, those with primary education (aRR 1.18, 95% CI: 1.12–1.23), secondary education (aRR 1.29, 95% CI: 1.22–1.36), and higher education (aRR 1.33, 95% CI: 1.24–1.42) all had significantly higher testing rates (all *p* < 0.0001).

#### Wealth index

Household wealth was significantly associated with testing (LRT *p* < 0.0001). Compared with the poorest quintile, women in the poorer (aRR 1.10, 95% CI: 1.01–1.19, *p* = 0.027), middle (aRR 1.15, 95% CI: 1.05–1.25, *p* = 0.0027), richer (aRR 1.24, 95% CI: 1.13–1.35, *p* < 0.0001), and richest (aRR 1.16, 95% CI: 1.05–1.27, *p* = 0.0023) quintiles all had higher testing rates.

#### Marital status

Marital status was significantly associated with HIV testing (LRT *p* < 0.0001). Compared with never-married women, those formerly married (aRR 1.39, 95% CI: 1.26–1.55), those who previously lived with a man (aRR 1.45, 95% CI: 1.36–1.54), and currently married or cohabiting women (aRR 1.44, 95% CI: 1.35–1.53) all had significantly higher testing rates (all *p* < 0.0001).

#### Healthcare utilization

Women who visited a health facility in the previous 12 months had significantly higher HIV testing uptake compared with those who had not (aRR 1.21, 95% CI: 1.16–1.26, *p* < 0.0001).

#### Internet use

Internet use was significantly associated with testing (LRT *p* = 0.0004). Compared with women who never used the internet, those who used it less than once a week (aRR 1.10, 95% CI: 1.03–1.16, *p* = 0.0031) and those who used it almost every day (aRR 1.05, 95% CI: 1.02–1.07, *p* = 0.0009) had significantly higher testing rates. Use at least once a week was not significantly associated with testing (*p* = 0.47).

#### Radio listening

Radio listening showed a significant overall association (LRT *p* = 0.043). Listening less than once a week was inversely and significantly associated with testing (aRR 0.94, 95% CI: 0.89–0.99, *p* = 0.031), while listening at least once a week was not significantly associated (*p* = 0.33).

#### Variables not independently associated with HIV testing

Religion (LRT *p* = 0.71), health insurance (LRT *p* = 0.38), newspaper or magazine reading (LRT *p* = 0.99), and television viewing (LRT *p* = 0.63) were not significantly associated with HIV testing after adjustment for other covariates.

Model diagnostics, including multicollinearity assessment, goodness-of-fit tests, overall model fit statistics, and the results of the reduced model (Model 2), are reported in the Supplementary File.

## Discussion

This study identified several sociodemographic and behavioral factors influencing HIV testing among women aged 15–49 years in Mozambique, using DHS data from 2022/23. Increasing age was strongly associated with HIV testing uptake, with all age groups older than 15–19 years demonstrating a significantly higher likelihood of testing. This association remained consistent in the multivariable analysis, where women aged 20–49 years had significantly higher adjusted risk ratios compared to those aged 15–19 years.

These findings align with a cross-sectional study conducted in Sub-Saharan Africa [15] and Mozambique2009 [16]. Similarly, studies in Kenya and Zambia reported that women aged 20–24 had the highest proportion of HIV testing [17]. A study in Tanzania also found that HIV testing rates were higher among individuals aged 18 and older (48.5%) compared to students younger than 18 (24.4%) [18].

Women aged 25–39 years typically exhibit higher levels of sexual activity, which increases their risk of HIV transmission. This risk is exacerbated by factors such as early sexual debut, inconsistent condom use, multiple sexual partners, and power imbalances in relationships. These elements not only elevate the likelihood of HIV transmission but also highlight the critical need for greater awareness of HIV risks and the importance of regular testing [19].

Moreover, women in this age group are more likely to access healthcare services for reproductive health, maternal care, or other medical needs. These frequent interactions with healthcare providers present valuable opportunities to offer HIV testing and counseling, ultimately encouraging greater testing uptake [20].This is supported by our analysis, which indicates that women who visited healthcare facilities in the past year were more likely to have undergone HIV testing. These findings are consistent with results from studies conducted in Eastern Africa [21], further emphasizing the importance of provider-initiated testing and counseling in improving testing uptake.

However, several barriers continue to limit healthcare utilization, including a lack of perceived risk, long distances to testing sites, and discomfort with the testing process [22–26].

Regarding type of place of resident, Urban women showed significantly higher HIV testing rates than rural women, likely due to better healthcare infrastructure, proximity to services, and exposure to awareness campaigns in urban areas. Conversely, rural populations face logistical challenges, such as long travel distances and limited healthcare resources, which act as barriers to testing [27, 28]. These findings aligned with previous studies in conducted in Sub-Saharan Africa [29], Eastern Africa [21] and Mozambique 2009 [16].

Moreover, women who resides in southern provinces showed significant better chances to perform HIV tests compared to those who resides in northern ones, particularly Niassa and Nampula. Such differences could be attributed to the better health infrastructure in southern provinces compared to northern ones [30, 31]. The findings align with reports from the civil society of the northern provinces considering public services and external support as insufficient resources [30]. The high illiteracy rates in Niassa (58%) and Nampula (56%), lower access to employment opportunities and lower response to poverty reduction policies in the northern region are other potential contributors to the current findings [31].

Education also plays a crucial role in influencing HIV testing. Higher levels of educational attainment are strongly associated with increased likelihood of undergoing HIV testing. These findings are consistent with the results of several previous studies [16, 32–34]. Education enhances health literacy, empowering women to seek healthcare services and making them more likely to understand the importance of HIV testing. Additionally, educated women tend to have greater autonomy and financial resources, which further enables their access to healthcare [28, 29].

Regarding wealth index, our analysis demonstrated a significant positive association with HIV testing uptake, whereby increasing household wealth was progressively associated with higher likelihood of testing. This finding aligns with evidence from sub-Saharan Africa and a prior study conducted in Mozambique in 2009, both of which reported that greater wealth was linked to increased HIV testing rates [16, 29]. Additionally, the absence of a significant association between health insurance coverage and HIV testing in our study may reflect the limited reach and low enrollment rates of formal health insurance schemes in Mozambique, where the majority of the population relies on out-of-pocket payments or donor-funded public health services. In this context, insurance status may not be a meaningful driver of access to HIV testing, which is largely offered free of charge through public health facilities and community-based programs. This finding suggests that financial protection mechanisms alone may be insufficient to explain testing behavior in low-income settings where universal HIV testing policies are in place.

Regarding exposure to mass media, our analysis found no significant association between the frequency of watching television, listening to the radio, or reading newspapers and magazines and the likelihood of HIV testing. However, frequent internet use was associated with higher HIV testing rates. These results align with a 2009 study in Mozambique, which found no significant association between watching television or listening to the radio and HIV testing [16]. In contrast, a study conducted in Sub-Saharan Africa found that individuals not exposed to radio, television, or newspapers were less likely to undergo HIV testing [29].

This suggests that traditional mass media channels may not be as influential in promoting HIV testing in Mozambique. It is possible that the content delivered through these channels is not as tailored or accessible to the specific health needs of the population. In contrast, the internet may provide more relevant, interactive, and personalized information, which could be more effective in motivating individuals to seek HIV testing.

Our findings emphasize the importance of considering the local context in public health interventions. The increasing use of the internet in Mozambique suggests that digital health campaigns, including social media and online resources, may be more effective in promoting HIV testing. Tailoring health messages to popular platforms can enhance engagement and improve health outcomes. Literature can benefit from future studies that adopt qualitative methodologies to further understand the underlying determinants of HIV testing. Also, future longitudinal studies will be beneficial to establish casual relationships.

### Strengths and limitations

This cross-sectional analysis used data from the 2022–2023 Mozambique Demographic and Health Survey (MDHS), which provides a comprehensive and nationally representative sample of women aged 15–49 years. The large sample size of 13,183 women strengthens the reliability of the findings and offers robust evidence on the sociodemographic and behavioral determinants of HIV testing, making it a valuable resource for informing targeted interventions and supporting national efforts to curb the HIV epidemic.

Despite these strengths, several limitations should be considered when interpreting the findings. First, cross-sectional design does not allow causal relationships to be established between the identified factors and HIV testing. In addition, the reliance on self-reported information may introduce recall bias, and social or cultural stigma surrounding HIV testing could result in underreporting. Furthermore, formal interaction analyses between key variables, such as region and healthcare access, wealth and education, were not conducted in this study. Future research employing interaction analyses and stratified approaches would provide more nuanced insights into how these factors jointly influence HIV testing uptake across different population subgroups in Mozambique. Furthermore, the analysis was restricted to variables available in the DHS dataset, meaning that potentially relevant factors such as HIV-related stigma, cultural beliefs, and perceptions of risk could not be examined.

With regard to the regression model, the primary model demonstrated good discriminatory performance, with an area under the curve (AUC) of 0.85, indicating a strong ability to distinguish between women who had and had not been tested for HIV. However, formal goodness-of-fit assessments suggested some degree of model miscalibration, with a tendency to underestimate HIV testing among women with lower predicted probabilities and overestimate it among those with higher predicted probabilities. It is important to note that the goodness-of-fit diagnostics applied in this study were originally developed and validated for standard non-survey samples and have not been formally extended to complex survey designs such as the DHS. Therefore, these calibration results should be interpreted cautiously and considered exploratory rather than definitive indicators of model fit.

## Conclusion

In conclusion, older age, living with a partner, higher educational attainment, greater household wealth, recent visits to health facilities, and frequent internet use were independently associated with a higher likelihood of HIV testing among women in Mozambique, whereas rural residence was associated with lower testing uptake. These findings highlight important disparities in HIV testing and emphasize the need for targeted interventions to improve access to testing in rural areas, reduce socioeconomic barriers, and expand the use of digital platforms for health promotion. Future longitudinal and qualitative studies are warranted to provide deeper insight into the underlying determinants of HIV testing and to inform more effective strategies for increasing testing coverage in this setting.

## Supplementary Information

Supplementary Material 1.

Supplementary Material 2.

## Data Availability

Data is available upon request from ICF International’s website ( https://dhsprogram.com/data/available-datasets.cfm ).
