# Supplementary material for: Factors influencing HIV testing among women in Mozambique: insights from the 2022/23 demographic and health survey
Source: BMC Public Health. 2026 Apr 28;26:1421. doi: 10.1186/s12889-026-27411-3 (PMC13130531; doi:10.1186/s12889-026-27411-3)
Supplement: Supplementary file 1 — Supplementary Material 1. [file 12889_2026_27411_MOESM1_ESM.docx]

**Supplementary file:**

**Supplementary Results**

Multicollinearity Assessment — Primary Model. Variance inflation was assessed using the Generalized Variance Inflation Factor (GVIF), with values adjusted for degrees of freedom (aGVIF = GVIF^1/(2·Df)^). All aGVIF values in the primary model were below 2.1 (range: 1.11–2.02), indicating no meaningful multicollinearity among the included variables (Supplementary Table S1). The highest value was observed for place of residence (aGVIF 2.02), likely to reflect its correlation with wealth index and region, but this remained within acceptable thresholds.

Model Fit — Primary Model. The primary model demonstrated good overall discriminatory ability, with an area under the receiver operating characteristic curve (AUC) of 0.85. The Cragg-Uhler pseudo-R² was 0.23 and the McFadden pseudo-R² was 0.19, indicating a moderate degree of variance explained. The Akaike Information Criterion (AIC) was 23,570.58.

Goodness-of-fit was assessed using two diagnostics adapted for modified Poisson regression by Hagiwara et al.: the modified Hosmer-Lemeshow test (T_mHL_ = 1429.80, df = 8, p<0.0001) and the Normalized Residual Sum of Squares test (statistic = 12.75, p<0.0001). Both tests rejected the null hypothesis of adequate calibration. Inspection of the group-level statistics revealed that the model underestimated testing in the lowest predicted probability group (observed: 257.70 vs. expected: 383.87) and overestimated in the highest group (observed: 1311.79 vs. expected: 1534.69), suggesting some miscalibration at the extremes of the predicted probability distribution. Importantly, these diagnostics were developed and validated for standard (non-survey) samples and have not been formally extended to complex survey designs; accordingly, these results should be interpreted as exploratory rather than definitive indicators of model fit.

Reduced Model Results (Model 2). A reduced model was fitted retaining variables with the strongest and most consistent associations: age (collapsed into three groups: 15–24, 25–34, and 35–49 years), place of residence, education, wealth index, marital status, health insurance, health facility visit in the last 12 months, and internet use. Region and religion were excluded from this model.

In the reduced model, all retained variables except health insurance remained significantly associated with HIV testing. Women aged 25–34 (aRR 1.17, 95% CI: 1.13–1.22) and 35–49 (aRR 1.16, 95% CI: 1.11–1.21) were more likely to have tested compared with those aged 15–24 (both p<0.0001). Rural residence remained inversely associated with testing (aRR 0.92, 95% CI: 0.88–0.95, p<0.0001). Compared with women with no education, those with primary (aRR 1.18, 95% CI: 1.13–1.24), secondary (aRR 1.30, 95% CI: 1.23–1.37), and higher (aRR 1.37, 95% CI: 1.28–1.46) education had significantly higher testing rates (all p<0.0001). Compared with the poorest quintile, women in the poorer (aRR 1.12, 95% CI: 1.03–1.22, p=0.0074), middle (aRR 1.21, 95% CI: 1.10–1.32), richer (aRR 1.34, 95% CI: 1.24–1.46), and richest (aRR 1.33, 95% CI: 1.22–1.45) wealth quintiles all had significantly higher testing (all p<0.0001 except poorer). Compared with never-married women, those formerly married (aRR 1.58, 95% CI: 1.42–1.76), those who previously lived with a man (aRR 1.71, 95% CI: 1.61–1.82), and currently married or cohabiting women (aRR 1.65, 95% CI: 1.56–1.75) had significantly higher testing rates (all p<0.0001). Recent health facility visit was also positively associated (aRR 1.30, 95% CI: 1.25–1.36, p<0.0001). For internet use, compared with non-users, those using it less than once a week (aRR 1.14, 95% CI: 1.07–1.21), at least once a week (aRR 1.08, 95% CI: 1.04–1.12), and almost every day (aRR 1.13, 95% CI: 1.10–1.16) all had significantly higher testing (all p<0.0001). Health insurance was the only retained variable not significantly associated with testing in this model (LRT p=0.077).

Multicollinearity Assessment — Reduced Model. All aGVIF values in the reduced model were below 1.30 (range: 1.09–1.30), confirming the absence of multicollinearity concerns after removal of region and religion (Supplementary Table S2).

Model Fit — Reduced Model. The reduced model showed slightly lower explanatory power compared with the primary model (Cragg-Uhler pseudo-R² = 0.19, McFadden pseudo-R² = 0.15, AIC = 23,753.54). The modified Hosmer-Lemeshow test (T_mHL_ = 1074.83, df = 8, p<0.0001) and Normalized Residual Sum of Squares test (statistic = 9.77, p<0.0001) similarly indicated poor calibration, with the same pattern of underestimation in lower and overestimation in higher predicted probability groups. As with the primary model, these findings should be interpreted with caution given that these diagnostics have not been validated for complex survey data.

**Supplementary analysis**

**1. Primary model specifications and fit statistics:**

**Supplementary Table S1: Variance inflation factor**

| **Variable** | **GVIF** | **Df** | **aGSIF** |
| --- | --- | --- | --- |
| age | 10.64 | 6.00 | 1.22 |
| region | 81.59 | 10.00 | 1.25 |
| residence | 4.07 | 1.00 | 2.02 |
| education | 5.12 | 3.00 | 1.31 |
| wealth | 16.20 | 4.00 | 1.42 |
| religion | 15.42 | 6.00 | 1.26 |
| marriage | 4.49 | 3.00 | 1.28 |
| insurance | 1.23 | 1.00 | 1.11 |
| visit | 1.58 | 1.00 | 1.26 |
| reading | 2.10 | 2.00 | 1.20 |
| radio | 2.91 | 2.00 | 1.31 |
| tv | 4.57 | 2.00 | 1.46 |
| internet | 2.23 | 3.00 | 1.14 |
| GVIF = Generalized Variance Inflation Factor , aGSIF = adjusted Generalized Standard Error Inflation Factor and is equal to GVIF^(1/(2*Df)) | | | |

**Modified Hosmer-Lemeshow Test for Modified Poisson Regression**

(These goodness-of-fit assessments (including the Normalized Residual Sum of Squares) were adopted from the paper by Hagiwara et al., <https://doi.org/10.1177/09622802241254220>. These diagnostics were developed and validated via simulation for standard (non-survey) samples. To our knowledge, such diagnostics have not been formally extended to complex survey designs. Accordingly, these tests should be interpreted as exploratory rather than definitive measures of model fit.)

Number of groups (g): 10
Test statistic (T_mHL): 1429.796
Degrees of freedom: 8
P-value: < 0.0001
Conclusion: Reject null hypothesis (poor model fit, p < 0.05)

Group-level statistics:

| group | n_j | O_j | E_j | pi_bar_j | S_j |
| --- | --- | --- | --- | --- | --- |
| 1 | 976 | 257.70 | 383.87 | 0.29 | 219.40 |
| 2 | 1243 | 521.63 | 536.65 | 0.41 | 315.53 |
| 3 | 1084 | 642.12 | 632.41 | 0.48 | 329.86 |
| 4 | 1116 | 754.55 | 706.61 | 0.54 | 323.59 |
| 5 | 1103 | 851.35 | 793.98 | 0.60 | 304.92 |
| 6 | 1212 | 955.50 | 885.54 | 0.67 | 265.50 |
| 7 | 1421 | 1113.09 | 993.91 | 0.75 | 184.40 |
| 8 | 1559 | 1218.28 | 1128.95 | 0.86 | 97.95 |
| 9 | 1700 | 1280.16 | 1309.56 | 0.99 | 38.19 |
| 10 | 1769 | 1311.79 | 1534.69 | 1.16 | 43.63 |

**Normalized Residual Sum of Squares Test for Modified Poisson Regression**

Test statistic: 12.75
P-value: < 0.0001
Conclusion: Reject null hypothesis (poor model fit, p < 0.05)
Residual Sum of Squares: 2116.373
Expected Variance Term: 2000.343

MODEL FIT:

*Pseudo-R² (Cragg-Uhler)* = 0.23
*Pseudo-R² (McFadden)* = 0.19
*AIC* = 23570.58
Area under the curve: 0.8516

2. Model 2 (reduced model):

| Variable | aRR*^a^* | 95% CI*^a^* | p-value | LRT p-value |
| --- | --- | --- | --- | --- |
| Age (years) |  |  |  | **<0.0001** |
| 15-24 | — | — |  |  |
| 25-34 | 1.17 | 1.13 to 1.22 | **<0.0001** |  |
| 35-49 | 1.16 | 1.11 to 1.21 | **<0.0001** |  |
| Place of residence |  |  |  | **<0.0001** |
| Urban | — | — |  |  |
| Rural | 0.92 | 0.88 to 0.95 | **<0.0001** |  |
| Highest educational level |  |  |  | **<0.0001** |
| No education | — | — |  |  |
| Primary | 1.18 | 1.13 to 1.24 | **<0.0001** |  |
| Secondary | 1.3 | 1.23 to 1.37 | **<0.0001** |  |
| Higher | 1.37 | 1.28 to 1.46 | **<0.0001** |  |
| Wealth index |  |  |  | **<0.0001** |
| Poorest | — | — |  |  |
| Poorer | 1.12 | 1.03 to 1.22 | **0.0074** |  |
| Middle | 1.21 | 1.10 to 1.32 | **<0.0001** |  |
| Richer | 1.34 | 1.24 to 1.46 | **<0.0001** |  |
| Richest | 1.33 | 1.22 to 1.45 | **<0.0001** |  |
| Ever been married or in a union |  |  |  | **<0.0001** |
| No | — | — |  |  |
| Formerly married | 1.58 | 1.42 to 1.76 | **<0.0001** |  |
| Lived with a man | 1.71 | 1.61 to 1.82 | **<0.0001** |  |
| Currently married/Lives with a man | 1.65 | 1.56 to 1.75 | **<0.0001** |  |
| Has health insurance |  |  |  | 0.077 |
| No | — | — |  |  |
| Yes | 0.96 | 0.91 to 1.00 | 0.076 |  |
| Visited a health facility in the last 12 months |  |  |  | **<0.0001** |
| No | — | — |  |  |
| Yes | 1.3 | 1.25 to 1.36 | **<0.0001** |  |
| Uses internet |  |  |  | **<0.0001** |
| Not at all | — | — |  |  |
| Less than once a week | 1.14 | 1.07 to 1.21 | **<0.0001** |  |
| At least once a week | 1.08 | 1.04 to 1.12 | **<0.0001** |  |
| Almost every day | 1.13 | 1.10 to 1.16 | **<0.0001** |  |
| *^a^ aRR = adjusted Risk Ratio, CI = Confidence Interval, LRT = Likelihood Ratio Test* | | | | |

**3. Model specifications and fit statistics (reduced model):**

**Supplementary Table S2:Variance inflation factor**

| **Variable** | **GVIF** | **Df** | **aGSIF** |
| --- | --- | --- | --- |
| age | 2.26 | 2 | 1.23 |
| residence | 1.68 | 1 | 1.30 |
| education | 2.15 | 3 | 1.14 |
| wealth | 2.80 | 4 | 1.14 |
| marriage | 1.93 | 3 | 1.12 |
| insurance | 1.19 | 1 | 1.09 |
| visit | 1.19 | 1 | 1.09 |
| internet | 1.65 | 3 | 1.09 |
| GVIF = Generalized Variance Inflation Factor , aGSIF = adjusted Generalized Standard Error Inflation Factor and is equal to GVIF^(1/(2*Df)) | | | |

**Modified Hosmer-Lemeshow Test for Modified Poisson Regression**

Number of groups (g): 10
Test statistic (T_mHL): 1074.829
Degrees of freedom: 8
P-value: < 0.0001
Conclusion: Reject null hypothesis (poor model fit, p < 0.05)

Group-level statistics:

| **group** | **n_j** | **O_j** | **E_j** | **pi_bar_j** | **S_j** |
| --- | --- | --- | --- | --- | --- |
| 1 | 1024 | 288.36 | 445.87 | 0.34 | 244.29 |
| 2 | 1087 | 563.05 | 578.57 | 0.44 | 322.58 |
| 3 | 1159 | 704.37 | 675.18 | 0.50 | 339.59 |
| 4 | 1183 | 733.12 | 705.90 | 0.55 | 314.69 |
| 5 | 1317 | 894.78 | 792.53 | 0.60 | 292.65 |
| 6 | 1352 | 970.14 | 903.23 | 0.66 | 285.34 |
| 7 | 1421 | 1089.50 | 996.94 | 0.74 | 213.25 |
| 8 | 1407 | 1118.28 | 1051.80 | 0.84 | 128.23 |
| 9 | 1663 | 1287.91 | 1297.39 | 0.97 | 53.98 |
| 10 | 1570 | 1256.66 | 1458.77 | 1.15 | 48.63 |

**Normalized Residual Sum of Squares Test for Modified Poisson Regression**

Test statistic: 9.7704
P-value: < 0.0001
Conclusion: Reject null hypothesis (poor model fit, p < 0.05)
Residual Sum of Squares: 2239.023
Expected Variance Term: 2145.028

**MODEL FIT:**

Pseudo-R² (Cragg-Uhler) = 0.19
Pseudo-R² (McFadden) = 0.15
AIC = 23753.54
Area under the curve: 0.8219
